# Impffortschritt in Deutschland und der Welt: Chancen und Risiken

**DOI:** 10.1007/s10273-021-2894-5

**Published:** 2021-04-17

**Authors:** Veronika Grimm, Franziska K. Lembcke, Milena Schwarz

**Affiliations:** 1 FB Wirtschafts- und Sozialwissenschaften, Friedrich-Alexander-Universität Erlangen-Nürnberg, Findelgasse 7/9, 90402 Nürnberg, Deutschland; 2 Statistisches Bundesamt, Gustav-Stresemann-Ring 11, 65189 Wiesbaden, Deutschland

**Keywords:** H4, H12, H51, I18

## Abstract

Deutschland hat sich das Ziel gesetzt, bis zum 21. September 2021 70 % der erwachsenen Bevölkerung ein Impfangebot zu unterbreiten. Der Fortschritt der Impfkampagne hängt dabei wesentlich von drei Determinanten ab: der Impfstoffverfügbarkeit, den Impfkapazitäten sowie der Impfbereitschaft. Auf Basis von Szenarienrechnungen wird aufgezeigt, wie das Ziel der Bundesregierung zu erreichen ist und welche Anforderungen an die Organisation der Impfkampagne dabei gestellt werden müssen. Abschließend werden langfristige Perspektiven und Herausforderungen angesprochen, etwa mit Blick auf die Impfung von Kindern und Jugendlichen oder den weltweiten Impffortschritt.

## Abstract (en)

The success of the vaccination campaign in Germany depends decisively on the availability of vaccines, vaccination capacities and the willingness to vaccinate. On the basis of model calculations, this article presents the possible scenarios of the vaccination progress and discusses requirements for the organisation of the vaccination campaign. It also addresses the long-term perspectives and challenges, e. g. with regard to the vaccination of children and adolescents or the global vaccination progress.

Mehr als ein Jahr nach Bekanntwerden der ersten Infektionen mit dem Virus SARS-CoV-2 hat die Pandemie Deutschland und viele Länder nach wie vor fest im Griff. Seit Ende Februar 2021 sieht sich Deutschland erneut mit einem Anstieg der Neuinfektionen konfrontiert (vgl. Abbildung 1 links). Durch das Auftreten verschiedener Virusvarianten besteht ein erhöhtes Risiko einer erneuten stärkeren Zunahme der COVID-19-Fälle (RKI, 2021c). Vor diesem Hintergrund ist die Verfügbarkeit von wirksamen Impfstoffen gegen COVID-19 und ein zügiger Impffortschritt von zentraler Bedeutung für die Bekämpfung des SARS-CoV-2-Virus und für eine Normalisierung des wirtschaftlichen und gesellschaftlichen Lebens.

Bislang haben in der EU zwei mRNA-Impfstoffe der Hersteller BioNTech/Pfizer und Moderna sowie Vektorimpfstoffe von AstraZeneca und Johnson & Johnson eine Zulassung als Impfung gegen COVID-19 erhalten. Aktuelle Studien deuten darauf hin, dass die Impfstoffe von BioNTech/Pfizer und Moderna die Zahl schwerer Infektionsverläufe reduzieren, auch solche, die durch die Mutationen B.1.1.7 und B.1.351 ausgelöst werden (Muik et al., 2021; Wang et al., 2021a; Xie et al., 2021; Dagan et al., 2021). Ebenso zeigen Studien, dass der Impfstoff von AstraZeneca schwere Verläufe, auch bei Älteren, reduziert (Voysey et al., 2021; Bernal et al., 2021) und ähnlich wirksam gegen die B.1.1.7-Variante wie gegen das ursprüngliche Virus ist (Emary et al., 2021). Die verfügbaren Impfstoffe könnten überdies an neue Varianten angepasst werden (Davies et al., 2021b; Tada et al., 2021; Volz et al., 2021). In Deutschland konnten bislang 10 % der Bevölkerung mindestens eine Impfdosis erhalten, während es weltweit rund 3,8 % waren (Stand 26. März 2021). Weltweit führend beim Impffortschritt ist Israel, wo bereits 60,2 % der Bevölkerung mindestens eine Impfdosis erhalten haben (26. März 2021, vgl. Abbildung 1 rechts). Während Großbritannien, Chile und die USA ebenfalls überdurchschnittlich viele Impfungen durchführen konnten, liegen die Mitgliedstaaten der EU sowohl bei den aktuell täglich verimpften Dosen als auch bei der Gesamtzahl der bereits verabreichten Impfdosen deutlich zurück. Deutschland liegt innerhalb der EU im Mittelfeld.

**Prof. Dr. Veronika Grimm** lehrt an der Friedrich-Alexander-Universität Erlangen-Nürnberg und ist Mitglied im Sachverständigenrat zur Begutachtung der gesamtwirtschaftlichen Entwicklung (SVR).

**Dr. Franziska K. Lembcke** und **Dr. Milena Schwarz** gehören dem wissenschaftlichen Stab beim SVR an.

Die EU hat das Ziel, bis zum Ende des Sommers 2021 mindestens 70 % der erwachsenen Bevölkerung der Mitgliedstaaten zu impfen (EU-Kommission, 2021a). Die Bundesregierung möchte allen Bürger:innen in Deutschland bis zum 21. September 2021 ein Impfangebot unterbreiten (Bundesregierung, 2021). Wie dieses Ziel zu erreichen ist und welche Kapazitätsengpässe sich auf diesem Pfad ergeben könnten, ist aufgrund des Zusammenhangs zwischen dem Infektionsgeschehen und der konjunkturellen Entwicklung (SVR, 2020; 2021) von hoher ökonomischer Relevanz. Nur durch einen zügigen Impffortschritt entstehen Perspektiven für eine umfangreiche Lockerung der gesundheitspo-

Abbildung 1

**Entwicklung der Neuinfektionen in Deutschland und Impffortschritt im internationalen Vergleich**

[1] Dem RKI übermittelte COVID-19-Fälle in Deutschland für die Meldewochen 10 2020 bis 11 2021 (Stand 23. März 2021). [2] Stand 19. März 2021. Die Todesfälle werden vom RKI mit einer Verzögerung von drei Wochen veröffentlicht. Für die Fälle bis KW 8 kann es dennoch zu Nachmeldungen kommen. [3] Anteil an der Gesamtbevölkerung. Entspricht nicht dem Anteil der vollständig geimpften Personen, da Impfstoffe zwei Dosen erfordern können (Stand 26. März 2021).

Quellen: Our World in Data; RKI.

litischen Einschränkungen und damit für eine nachhaltige wirtschaftliche Erholung. Gleichzeitig stellt die Frage, wie sich die Verteilung von knappen (Gesundheits-)Gütern effizient und bedarfsgerecht organisieren lässt, eine thematische Kernkompetenz von Ökonom:innen dar.

Eine besondere Dringlichkeit des Impffortschritts ergibt sich durch Virusmutationen. Derzeit verbreiten sich neue Virusvarianten (B.1.1.7, B.1.351 und P.1) in verschiedenen Ländern sehr dynamisch. Alle drei Varianten wurden bereits in Deutschland nachgewiesen, und seit Anfang 2021 ist speziell der Anteil der Variante B.1.1.7, die mit einer erhöhten Übertragbarkeit des Virus einhergeht, an den nachgewiesenen Neuinfektionen deutlich gestiegen (RKI, 2021d). Zudem dürften die Infektionen mit der Variante B.1.1.7 mit einer erhöhten Fallsterblichkeitsrate einhergehen (Challen et al., 2021; Davies et al., 2021a).

## Impfstofflieferungen und verimpfte Dosen

In Deutschland konnten bislang rund 4,4 % der Bevölkerung vollständig geimpft werden, 10 % der Bevölkerung haben mindestens eine Impfdosis erhalten (Stand 26. März 2021). Seit Anfang März 2021 wurden in Deutschland durchschnittlich über 200.000 Impfungen pro Tag durchgeführt, mit steigender Tendenz. Im Wochenverlauf kommt es jedoch immer wieder zu Schwankungen der verabreichten Impfdosen, da an den Wochenenden bisher weniger geimpft wird. Zuletzt kam es zudem zu einem Abfall der Werte aufgrund einer temporären Aussetzung der Verimpfung von AstraZeneca vom 15. bis zum 19. März (BMG, 2021b; PEI, 2021) (vgl. Abbildung 2 links).

Während Anfang 2021 die Verfügbarkeit von Impfstoffen aufgrund der zunächst beschränkten Liefermengen gering war, dürfte Deutschland ab dem zweiten Quartal Zugang zu deutlich mehr Impfstoff erhalten (vgl. Abbildung 2 rechts). Das BMG prognostiziert allein für den Zeitraum vom 5. April bis 2. Mai 2021 eine Liefermenge von insgesamt 15,2 Mio. Impfstoffdosen (MPK, 2021b). Dies entspricht etwa der Menge, die seit Beginn der Impfkampagne im Dezember 2020 in Deutschland bislang geliefert wurde (BMG, 2021c). Auf Basis der vertraglich zugesicherten Liefermengen der COVID-19-Impfstoffe dürften bis zum Ende des dritten Quartals insgesamt 217 Mio. Impfdosen zur Verfügung stehen. Nur ein kleiner Teil (10,8 Mio. Impfdosen) entfällt dabei auf Impfstoffkandidaten in noch laufenden klinischen Prüfungen und hängt von einer erfolgreichen Zulassung ab. Mit den bereits zugelassenen Impfstoffen könnten rein rechnerisch mit der bis zum Ende des dritten Quartals zur Verfügung stehenden Menge von 206,2 Mio. Impfdosen rund 119 Mio. Erwachsene vollständig geimpft werden, wobei berücksichtigt ist, dass bei dem Vakzin von Johnson & Johnson nur eine Dosis, bei den anderen Vakzinen zwei Dosen für einen vollständigen Schutz verimpft werden müssen. In Deutschland müssten für die Durchimpfung von 70 % der erwachsenen Bevölkerung rund 48,6 Mio. Menschen geimpft werden. Demnach steht theoretisch bis zum Ende des dritten Quartals ausreichend Impfstoff zur Verfügung, um das EU-Ziel zu erfüllen.

Abbildung 2
**Verimpfte Dosen und Impfstofflieferungen in Deutschland**

[1] Stand 26. März 2021. [2] Noch keine Zulassung durch die Europäische Arzneimittel-Agentur (EMA).

Quellen: BMG; RKI; Zentralinstitut für die kassenärztliche Versorgung (ZI).

Gleichwohl besteht das Risiko, dass es im Jahresverlauf trotz vertraglicher Zusagen noch zu einer Verzögerung der Lieferungen durch die Hersteller kommen kann. Die Liefertermine und -mengen sind mit einer Reihe von produktionsbedingten Unsicherheiten behaftet, etwa mit den bei einigen Impfstoffkandidaten noch laufenden klinischen Prüfungen, von erfolgreich abzuschließenden Zulassungsverfahren, der Stabilität der Produktionsprozesse und Lieferketten für Vorprodukte sowie von erfolgreichen Qualitätskontrollen (Kirchhoff, 2021). Bereits im ersten Quartal war es zu Lieferengpässen einzelner Hersteller gekommen (Ärztezeitung, 2021; Deutsches Ärzteblatt, 2021), ein solches Szenario ist auch für den weiteren Jahresverlauf möglich. Zwar würde die Menge der im Jahresverlauf durch die anderen Hersteller vertraglich zugesagten Impfstoffdosen theoretisch sogar ausreichen, um mögliche Liefer- oder Chargenausfälle eines einzelnen Vakzins gänzlich zu kompensieren. Gleichwohl stellen Lieferverzögerungen, gerade vor dem Hintergrund der in mehreren Ländern verhängten Exportbeschränkungen für COVID-19-Impfstoffe, aktuell eine der größten Herausforderungen für den Fortschritt der Impfkampagne in Deutschland dar.

Die EU verfolgt eine europäische Strategie zur Entwicklung, Herstellung und Bereitstellung von Impfstoffen gegen COVID-19 (EU-Kommission, 2020). Die EU-Kommission konnte sich kürzlich durch Nachverhandlungen mit BioNTech/Pfizer zusätzlich 4 Mio. Impfdosen für Corona-Hotspots für März 2021 sichern, wovon 580.000 Dosen auf Deutschland entfallen (EU-Kommission, 2021h; MPK, 2021b). Zudem hat die EU mit BioNTech/Pfizer eine vorgezogene Lieferung von 10 Mio. Impfdosen im zweiten Quartal vereinbart (EU-Kommission, 2021j). Aus dem Teil der EU-Verträge mit den Impfstoffherstellern, die öffentlich einsehbar sind (EU-Kommission, 2021b, 2021c, 2021d), lässt sich ablesen, dass die EU mit den Herstellern Verträge mit vagen Lieferzeiten vereinbart hat, die in Quartalen angegeben sind. Zudem scheinen die Verträge kaum Vorkehrungen für Lieferverzögerungen zu enthalten. Angesichts der massiven gesellschaftlichen und ökonomischen Kosten, die eine Verzögerung um einen oder zwei Monate hätte, dürften solche Vereinbarungen unzureichende Anreize für eine kurzfristige Ausdehnung der Produktionskapazitäten setzen (Wambach und Gretschko, 2021). Zudem erfordern solch vage Zeitfenster ein hohes Maß an Flexibilität hinsichtlich der Anpassung der Kapazitäten an die Liefermengen. Um den Umgang mit einer Regulierung von Exporten von COVID-19-Impfstoffen wird aktuell innerhalb der EU gerungen. Seit dem 1. Februar 2021 müssen Impfstoff-Exporte aus EU-Staaten in viele Länder angemeldet und genehmigt werden (EU-Kommission, 2021). Jüngst beschloss die EU-Kommission, dass der COVID-19-Impfstoff-Export, mit deren Herstellern EU-Verträge bestehen, künftig noch strenger kontrolliert und notfalls gestoppt werden soll (EU-Kommission, 2021i). Bei der Bewältigung der Pandemie und angesichts der langfristigen Herausforderungen ist internationale Arbeitsteilung und Kooperation essenziell. Sollte es zu weiteren Exportbeschränkungen für COVID-19-Impfstoffe durch die EU kommen, könnten diese von wichtigen Handelspartnern als protektionistische Maßnahme gewertet werden und zu Gegenmaßnahmen führen. Dies könnte deswegen wenig zielführend sein, da die EU bei der Impfstoffproduktion auf Importe angewiesen sein dürfte. Für die EU, die nicht nur im Kontext von medizinischen Gütern überdurchschnittlich stark in globale Lieferketten eingebunden ist (Fries et al., 2020), kann ein Handelskonflikt zudem mit einem signifikanten Verlust an Wertschöpfung einhergehen. Vor diesem Hintergrund sind die Risiken durch Handelsbarrieren gegen eine mögliche weitere Verzögerung der Impfkampagne durch einen Impfstoffmangel in der EU abzuwägen.

Um den Herstellern einen größeren Anreiz für eine schnellere Produktion zu bieten, könnten stattdessen marktbasierte Anreize hilfreich sein. Hierbei kann es um neue Verträge mit den Herstellern, zu einem höheren Preis je Impfdosis, und somit eine Aufstockung der bisher vertraglich vereinbarten Liefermengen gehen. Nachverhandlungen der bestehenden Verträge mit entsprechend höheren preislichen Anreizen könnten aber auch sicherstellen, dass bereits bestellte Impfstoffe zügig geliefert werden. Die EU hat bislang vertraglich insgesamt 2,6 Mrd. Dosen für ihre 450 Mio. Bürger:innen sicherstellen können. Daher geht es nun hauptsächlich darum, dass die bereits bestellten und vertraglich zugesicherten Impfdosen Deutschland so zeitnah wie möglich zur Verfügung stehen. Die EU könnte den Herstellern etwa zusätzliche Prämien für jede vorzeitig gelieferte Dosis zahlen (Fuest und Gros, 2021). Unter diesen Voraussetzungen könnten Unternehmen aus ihrem privatwirtschaftlichen Kalkül entscheiden, ob sie weitere Produktionskapazitäten aufbauen, ihre Kooperationsbemühungen verstärken oder zusätzliche Lizenzen an Auftragsfertiger vergeben. Eine Studie von Ahuja et al. (2021) zeigt, dass die Pandemie die weltweite Wirtschaftsleistung pro Monat um rund 420 Mrd. Euro reduziert und dass jede Dosis Impfstoff, die 2021 geliefert wird, einen Nutzen für die Gesellschaft von etwa 1.500 Euro hat. Entsprechend groß dürfte sich der finanzielle Spielraum für solche Prämien bemessen lassen. Zusätzlich oder alternativ könnte den Herstellern eine Prämie für die Auslieferung an festgelegten Terminen angeboten werden, um die Planbarkeit für die weitere Impfkampagne zu erhöhen. Bei entsprechender Ausgestaltung (Fuest und Gros, 2021), könnten diese einen starken Anreiz für die Unternehmen schaffen, die Produktion zu beschleunigen. Im Zeitablauf mit einer steigenden Impfquote der Bevölkerung könnten die Prämien dann abgesenkt werden. Kirchhoff (2021) kritisiert, dass durch Prämien ein Überbietungswettbewerb zwischen Staaten ausgelöst werden könnte, der insbesondere schädlich für die ohnehin schon relativ benachteiligten ärmeren Länder wäre. Ziel sollte jedoch nicht sein, durch preisliche Anreize Renten zum Nachteil anderer Länder abzuschöpfen, sondern den Herstellern einen zusätzlichen Anreiz für eine Ausweitung der Produktionskapazitäten zu schaffen. Durch eine solche Ausweitung könnten letztlich positive Externalitäten für alle Länder entstehen. Einerseits kann dadurch auch für andere Länder eine frühe Lieferung von bestellten Vakzinen ermöglicht werden und andererseits die Welt insgesamt von einer beschleunigten Impfkampagne profitieren (Ahuja et al., 2021).

## Impfkapazitäten und Kapazitätsauslastung

In Deutschland wird aktuell hauptsächlich in Impfzentren sowie dezentral durch mobile Teams geimpft. Es existieren derzeit 433 Impfzentren, die sich über alle Bundesländer verteilen. Nach Angaben des Zentralinstituts für die kassenärztliche Versorgung (ZI) liegen die gemeldeten maximalen Impfkapazitäten in den Impfzentren derzeit bei rund 340.000 Impfungen pro Tag (Stand 24. März 2021), die aktuell jedoch nicht vollständig ausgeschöpft werden, da bislang noch zu wenig Impfstoffdosen zur Verfügung stehen.

Mit zunehmender Impfstoffverfügbarkeit werden nicht mehr die Zahl der Impfdosen, sondern die Impfkapazitäten der limitierende Faktor sein. Für eine Erhöhung der täglichen Impfungen stehen verschiedene Kanäle zur Verfügung. Es dürften in den bundesweiten Impfzentren deutliche Potenziale bestehen, die dort täglich durchgeführten Impfungen zu steigern, etwa über eine Ausweitung der Öffnungszeiten sowie durch mehr Impfungen am Wochenende. Um die Impfprozesse zudem weiter zu beschleunigen, möglichst viele Menschen zu mobilisieren und verwaltungstechnische Hürden im Bereich Registrierung und Terminkoordination abzubauen, ist die Nutzung von bestehender Gesundheitsinfrastruktur durch haus- und fachärztliche Praxen sowie eine Einbindung von Betriebsärzt:innen zielführend. Dabei ist zentral, dass der Verwaltungs- und Dokumentationsaufwand in den Praxen einer zügigen Verimpfung nicht im Wege steht. Im Bund-Länder-Beschluss vom 19. März 2021 hat dies bereits entsprechende Berücksichtigung gefunden (MPK, 2021b). Für das Impfen in Praxen sind insbesondere Impfstoffe geeignet, die sich bei Kühlschranktemperatur lagern lassen. Unter den aktuell in der EU zugelassenen Impfstoffen trifft dies auf die Vakzine von AstraZeneca sowie Johnson & Johnson zu. Grundsätzlich erlauben auch die mRNA-Impfstoffe eine kurzzeitige Lagerung in den Praxen. Durch die deutlich geringere Haltbarkeit ergeben sich hierbei jedoch größere Herausforderungen bei der Vergabe von Impfterminen innerhalb der Praxis. Aktuell hat die Europäische Arzneimittelagentur (EMA) zudem die Lagerung des Vakzins von BioNTech/Pfizer bei -15° C genehmigt (EMA, 2021). Dies könnte den Transport des Vakzins und die Impfung in Praxen erleichtern.

In Deutschland wird zwischen sechs Priorisierungsgruppen unterschieden, die gemäß der Coronavirus-Impfverordnung und dem Stufenplan der Ständigen Impfkommission (STIKO) grundsätzlich nacheinander geimpft werden sollen (BMG, 2021a; RKI, 2021a). Aktuell wird Personen aus der zweiten Priorisierungsgruppe ein Impfangebot gemacht (MPK, 2021a). In Deutschland wurden bislang 47,6 % der Gesamtdosen an Personen mit Altersindikation verabreicht, während 40,9 % an Personen mit beruflicher Indikation – wie z. B. medizinisches Personal – gingen, 13,9 % an Pflegeheimbewohner:innen sowie 5,4 % an Personen mit Vorerkrankungen (26. März 2021). Dabei können Geimpfte mehreren Indikationen zugeordnet werden.

Die modelltheoretische Literatur zur optimalen altersspezifischen Verteilung von Impfstoffen verweist darauf, dass die prioritäre Impfung älterer Kohorten grundsätzlich im Sinne einer Eindämmung von schweren Verläufen und Todesfällen in der Pandemie sein dürfte. Die zukünftigen Inzidenzwerte dürften jedoch maßgeblich von der Impfquote unter (jüngeren) Menschen mit besonders vielen, auch generationenübergreifenden Kontakten abhängen (Wang et al., 2021b; Bubar et al., 2021). Im weiteren Verlauf der Impfkampagne könnte die Identifikation und gezielte Ansprache von Personen aus einzelnen Priorisierungsgruppen den Impfprozess zudem deutlich verlangsamen. Insbesondere existieren in Deutschland im Vergleich zu anderen Ländern keine Register zu Personen mit bestimmten Vorerkrankungen. Auch vor diesem Hintergrund ist eine Einbeziehung von niedergelassenen Ärzt:innen, die über einen entsprechenden Informationsvorsprung verfügen, besonders sinnvoll. Das Impfen ganzer Berufsgruppen gegen COVID-19 dürfte hingegen in Impfzentren effizienter sein als in Praxen.

Mit Blick auf das Impftempo sollte unbedingt sichergestellt werden, dass verfügbarer Impfstoff zeitnah verimpft wird und dass kurzfristige Impfterminausfälle schnellstmöglich kompensiert werden. Hierfür ist es zielführend, die Terminvergabe so transparent und einfach wie möglich zu gestalten. Eine verbesserte Organisation der Impflogistik könnte etwa mehrere Priorisierungsgruppen im Rahmen eines digital organisierten Registrierungs- und Nachrückverfahrens effizienter mit in den Impfprozess einbeziehen, damit täglich die nicht verabreichten Impfdosen schnellstmöglich verimpft werden können. Überdies könnte eine zielgenaue Impfstoff-Koordination mit Hilfe einer geeigneten Software für das Impfmanagement für mehr Planungssicherheit sorgen und somit die Impfkampagne beschleunigen. So könnte ein bundesweites Impfmanagementsystem zentral erfassen, wie viele Dosen in welche Impfzentren geliefert werden und ob Nachlieferungen nötig sind. Mit einer Einbeziehung von Arztpraxen und somit von bestehenden Warenwirtschaftssystemen, also über den Großhandel und die Apotheken, die eine Nachverfolgung des Impfstoffs in der Lieferkette ermöglichen, dürfte die Koordination ebenso an Effizienz gewinnen.

Eine Herausforderung bei der Impfstoffzuteilung an Arztpraxen kann sich außerdem dadurch ergeben, dass die Zahl der Arztpraxen nicht proportional zur Bevölkerung in den Bundesländern ist und sich darüber hinaus in den Städten die Arztpraxen möglicherweise nicht gleichmäßig auf die Stadtviertel verteilen. Die dadurch entstehende Herausforderung bei der Impfstoffverteilung an die Praxen könnte mit Hilfe einer entsprechend entwickelten Optimierungssoftware gelingen, die über Nebenbedingungen verschiedene zu beachtende Restriktionen betrachtet. Hierbei geht es einerseits um eine flächendeckende Koordination von Impfstoffverteilung und Terminen in den Praxen, die aufgrund der begrenzten Haltbarkeit der Vakzine dann (sofern der Großhandel die Ampullen aufgetaut an die Apotheken ausliefert) in einem begrenzten Zeitfenster an geeignete Patient:innen der jeweiligen Praxis innerhalb der aktuell angesprochenen Priorisierungsgruppe verimpft werden müssen. Andererseits könnte die Software zu einer gerechten Impfstoffverteilung auf Zentren und Praxen beitragen und dadurch die Schnelligkeit von Entscheidungen und deren Akzeptanz positiv beeinflussen.

## Impfbereitschaft

Nach aktuellen Umfragen geben rund 80 % der Deutschen an, sich „auf jeden Fall“ (66,8 %) oder „eher“ (13,2 %) gegen COVID-19 impfen lassen zu wollen (RKI, 2021b). Bei rund 70 Mio. Erwachsenen in Deutschland ergäben sich so 56 Mio. Impfwillige. Der Impfbereitschaft wird eine entscheidende Rolle für den Erfolg der Impfkampagne zukommen. Insgesamt kann davon ausgegangen werden, dass die Impfung mit den aktuell in Deutschland zugelassenen Impfstoffen einen entscheidenden Beitrag zur Eindämmung der Pandemie leisten kann, da hierdurch eine relevante Bevölkerungsimmunität ausgebildet wird (RKI, 2021g). Mit einer weit fortgeschrittenen Impfkampagne dürften die Herausforderungen für das Erreichen von potenziell Impfwilligen steigen. Aufgrund des bestehenden Vertrauensverhältnisses könnte sich grundsätzlich die Impfbereitschaft durch die Einbindung der Praxen steigern lassen. In der Modellrechnung wird davon ausgegangen, dass jeweils eine entsprechende Impfbereitschaft besteht, mit der das EU-Ziel erreichbar wäre. Mit Blick in die nähere Zukunft ist jedoch zu beachten, dass mit zunehmendem Impffortschritt die Ansprache und Motivation der Menschen immer wichtiger wird, da von einer im Schnitt immer geringeren Impfbereitschaft der ungeimpften Bevölkerung auszugehen ist. Auch aus diesem Grund müssen die Verfahren mit zunehmendem Impffortschritt stetig verbessert und weiterentwickelt werden.

## Kann das Ziel bis September 2021 erreicht werden?

Angesichts der dritten Corona-Welle ist ein zügiges Fortschreiten der Impfkampagne in Deutschland wichtig. Hierfür ist eine optimale Ausnutzung sowie Ausweitung der Impfkapazitäten (vor allem hinsichtlich steigender geplanter Liefermengen ab dem zweiten Quartal 2021) zentral, um den Impffortschritt zu beschleunigen und bis zum Ende des dritten Quartals, entsprechend dem EU-Ziel, 70 % der erwachsenen Bevölkerung gegen COVID-19 geimpft zu haben.[1]

Auf Basis einer Modellierung des ZI lassen sich verschiedene Szenarien zu Dauer und Umfang der Impfkampagne in Deutschland nach jeweils aktuell verfügbaren Informationen darstellen. Die Modellrechnung erlaubt zudem einen Abgleich zwischen den vertraglich zugesagten Impfdosen im Jahresverlauf sowie den jeweils verfügbaren Impfkapazitäten. In einem Basisszenario, in dem täglich 216.500 durchgeführte Impfungen unterstellt werden (500 Patient:innen pro Tag in jedem der 433 Impfzentren), die an 7 Tagen pro Woche stattfinden würden, wäre ohne Beschleunigung der Impfkampagne eine vollständige Impfung von 70 % der erwachsenen Bevölkerung in Deutschland Ende November 2021 erreicht (vgl. Abbildung 3 links). Das Ziel, bis zum 21. September 2021 allen Bürger:innen in Deutschland ein Impfangebot zu unterbreiten, würde nicht erreicht werden.

Das Impftempo, das von durchschnittlich rund 200.000 Impfungen pro Tag in den ersten Märzwochen zuletzt (in KW12) schon auf durchschnittlich 270.000 Impfungen pro Tag angestiegen ist, muss also in Zukunft deutlich gesteigert werden. Es stehen dafür mit den steigenden Impfstofflieferungen ab dem zweiten Quartal theoretisch ausreichend Impfstoffdosen zur Verfügung. Die erwarteten Impfstofflieferungen dürften dann die gemeldeten maximalen Kapazitäten in den bundesweiten Impfzentren sogar übersteigen (Differenz < 0 im Basisszenario) (vgl. Abbildung 3 rechts). Durch eine Kapazitätenausweitung sollte sich also eine deutliche Beschleunigung des Impffortschritts realisieren lassen. Im Folgenden wird durch eine Szenarienrechnung aufgezeigt, wie durch eine optimale Auslastung der Kapazitäten und die Einbeziehung von Haus-, Fach- und Betriebsärzt:innen das Impfgeschehen beschleunigt werden könnte. Gelingt es, die täglich verabreichten Dosen in den bundesweiten Impfzentren um 50 % zu steigern (Szenario 1), könnte eine vollständige Impfung von 70 % der erwachsenen Bevölkerung in Deutschland im September 2021 erreicht werden (vgl. Abbildung 3 links). Um eine solche Steigerung zu realisieren, müssten die aktuell maximal dem ZI gemeldeten Kapazitäten der Impfzentren fast vollständig ausgelastet werden (324.750 Impfungen täglich in der Modellrechnung, Szenario 1). Dennoch würde die im Rahmen der Kapazitäten der Impfzentren mögliche Beschleunigung des Impfgeschehens nicht ausreichen, um alle erwarteten Impfstofflieferungen zeitnah zu verimpfen (Differenz < 0 in Szenario 1, vgl. Abbildung 3 rechts). Schon ab Anfang April wäre modelltheoretisch mehr Impfstoff verfügbar als in den Impfzentren sogar bei fast vollständiger Auslastung verimpft werden könnte.

Im Jahresverlauf dürfte eine deutliche Beschleunigung der Impfkampagne daher nur unter Einbeziehung von Haus-, Fach- und Betriebsärzt:innen zu erzielen sein, die eine niedrigschwellige Parallelstruktur zu den bestehenden Impfzentren und mobilen Impfteams erlauben. Unter der Annahme, dass es gelingt, die tägliche Zahl an Impfungen in den Impfzentren um 50 % zu steigern und darüber hinaus Praxen (mit 20 Patient:innen pro Tag in 55.000 Arztpraxen, je 50.000 Haus- und Facharztpraxen und 5.000 auf Betriebsebene) in den Impfprozess miteinzubeziehen (Szenario 2), könnte Deutschland das 70 %-Ziel der EU bereits im Juli 2021 erreichen (vgl. Abbildung 3 links).

Bei der Frage, wann optimalerweise Praxen in den Impfprozess einbezogen werden sollten, sind grundsätzlich verschiedene Aspekte zu beachten. Es gilt durch den Einbezug der Praxen zu einer Beschleunigung der Impfkampagne beizutragen, aber auch um eine optimale Ausnutzung der verfügbaren Kapazitäten, damit verfügbarer Impfstoff jederzeit zügig verimpft wird. Darüber hinaus könnten weitere gewichtige Gründe für eine frühzeitige Einbindung der Praxen bestehen. Erst zum Zeitpunkt der Durchimpfung von Priorisierungsgruppe 3, Mitte Mai, würde sich modelltheoretisch durch die Parallelstruktur zwischen Praxen und Impfzentren eine deutliche Beschleunigung der Impfkampagne erreichen lassen (vgl. Abbildung 3 links). Dies ist darauf zurückzuführen, dass im Modell ein linearer Anstieg der Produktion und wöchentlichen Liefermengen der Vakzine im zweiten Quartal unterstellt wird. Diese Annahme ist allerdings mit Unsicherheit behaftet, da die vertragliche Zusicherung der Hersteller sich nur auf eine Gesamtmenge auf Quartalsbasis bezieht (vgl. Abbildung 2 rechts). Gleichwohl führt auch im Modell die Einbeziehung der Arztpraxen bereits ab Anfang April zu einer besseren Auslastung der verfügbaren Impfkapazitäten (vgl. Abbildung 3 rechts). Daher sollte jetzt, selbst wenn kurzfristig noch keine ausreichende Menge an Impfstoff zur Verfügung steht, um die Impfkampagne durch eine breite Einbindung von Praxen maßgeblich zu beschleunigen, eine schrittweise Einbindung von haus- und fachärztlichen Praxen sowie Betriebsärzt:innen, wie auf der

1 Die jüngste Empfehlung der STIKO vom 30.3.2021, den Impfstoff von AstraZeneca nur noch für über 60-Jährige zu verwenden (RKI, 2021h), dürfte eine Umorganisation der bereits vergebenen Termine erforderlich machen und das Tempo in den Impfzentren kurzfristig drosseln. Die Zielerreichung der Impfkampagne dürfte jedoch weder grundsätzlich gefährdet sein, noch sollte sich durch diese Entwicklung der Zeitpunkt, an dem Deutschland das 70%-Ziel der EU erreichen kann, wesentlich nach hinten verschieben. Dies gilt insbesondere, da spätestens ab dem dritten Quartal theoretisch ausreichend andere Vakzine zur Verfügung stehen dürften. Jedoch hat diese neueste Entwicklung in der Szenarienrechnung noch keine Berücksichtigung finden können.

Abbildung 3

**Modellierung verschiedener Szenarien zum Impffortschritt und zur Kapazitätsauslastung**

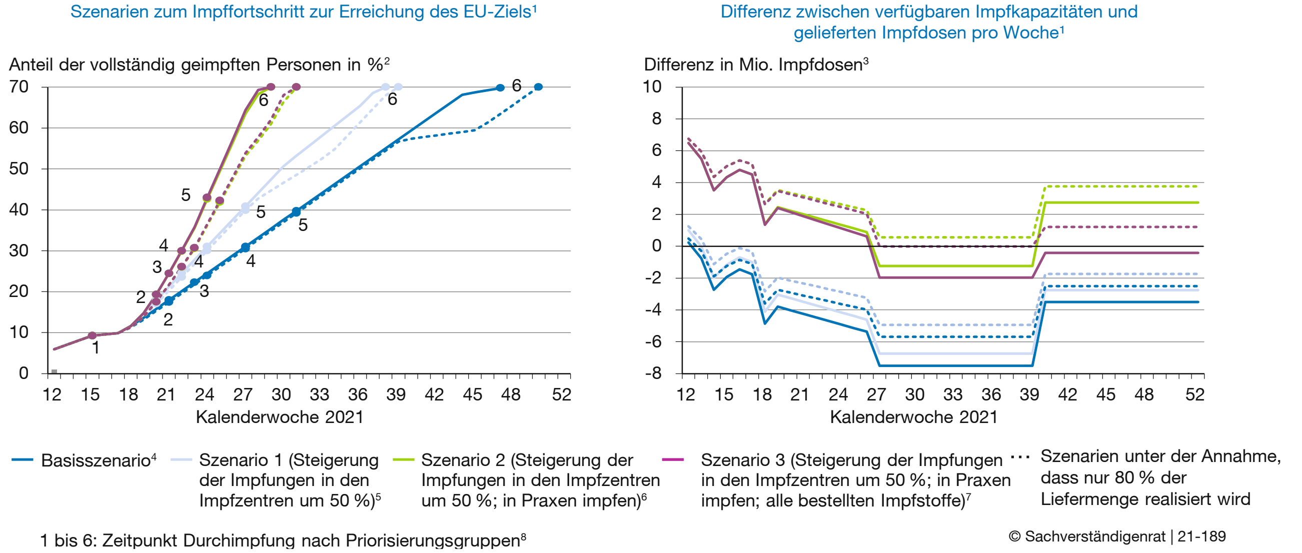

[1] 26. März 2021. [2] Anteil an der Bevölkerung ab 18 Jahren. Bei Erreichen einer Impfquote von 70 % im Sommer 2021 wäre das EU-Ziel erreicht. [3] Differenz zwischen verfügbaren Impfkapazitäten und gelieferten Impfdosen pro Woche. Impfkapazitäten entsprechen der möglichen durchführbaren Zahl an Impfungen pro Woche. [4] 433 Impfzentren, 500 Patient:innen pro Tag und Impfzentrum, sieben Arbeitstage pro Woche; keine Praxen; Liefermenge 100 %, Rückstellung für Zweitimpfungen (ja), Abstand Erst- zu Zweitimpfungen laut Ständiger Impfkommission (STIKO), nur zugelassene Impfstoffe, linearer Anstieg der Produktion in Q2, Impfbereitschaft 70 %. [5] 750 Patient:innen pro Tag und Impfzentrum. [6] 750 Patient:innen pro Tag und Impfzentrum; Impfungen in 55.000 Arztpraxen an fünf Arbeitstagen pro Woche; 20 Patient:innen pro Tag. [7] 750 Patient:innen pro Tag und Impfzentrum; Impfungen in 55.000 Arztpraxen an fünf Arbeitstagen pro Woche; 20 Patient:innen pro Tag; alle bestellten Impfstoffe. [8] Zahlen 1 bis 6 geben Zeitpunkt der Durchimpfung der Impfwilligen in der jeweiligen Priorisierungsstufe 1 bis 6 an. Einteilung der Priorisierungsstufen nach ZI laut STIKO-Empfehlung. Die Priorisierungsstufe 1 nach STIKO entspricht §2 (Höchste Priorität) der Impfverordnung; Priorisierungsstufen 2 und 3 nach STIKO entsprechen §3 (hohe Priorität) der Impfverordnung; Priorisierungsstufen 4 und 5 nach STIKO entsprechen §4 (erhöhte Priorität) der Impfverordnung des Bundesministeriums für Gesundheit.

Quelle: Zentralinstitut für die kassenärztliche Versorgung (ZI).

Bund-Länder-Konferenz vom 3. März 2021 beschlossen, unbedingt erfolgen (MPK, 2021a). Nur ein früher Einbezug von Arztpraxen erlaubt es, mögliche Probleme beim Hochlauf zu identifizieren und nachjustieren zu können. Zudem ermöglicht ein langsamer Einstieg frühzeitig die Identifikation und gezielte Ansprache von Impfwilligen aus der Priorisierungsgruppe 3.

Es besteht prinzipiell das Risiko, dass es im Jahresverlauf 2021 noch zu einer Verzögerung der vertraglich zugesicherten Lieferungen kommen kann. Dies könnte die skizzierte Beschleunigung der Impfkampagne durch eine Ausweitung der Kapazitäten gefährden. Um die bestehende Unsicherheit von Lieferengpässen oder Chargenausfällen in der Modellierung der notwendigen Impfquote zu adressieren, zeigt Abbildung 3 auch eine Szenarienrechnung unter Simulation eines Liefermengenausfalls von 20 %. Im Vergleich zur vorherigen Szenarienrechnung, in der angenommen wurde, dass die geplanten Liefermengen vollständig eintreffen, führt der modellierte Liefermengenausfall nur zu einer begrenzten Verzögerung von ein bis zwei Kalenderwochen. Das EU-Ziel würde in den Szenarien 1 bis 3 daher trotzdem erreicht (vgl. Abbildung 3 links). Zentraler Faktor für die Beschleunigung der Impfkampagne in Deutschland bleibt daher die Ausweitung und optimale Ausnutzung der Impfkapazitäten.

## Langfristige Perspektiven und Risiken

Laut Robert Koch-Institut (RKI) weisen nach aktuellem Kenntnisstand alle in der Europäischen Union zugelassenen Impfstoffe einen guten individuellen Schutz vor der Erkrankung an COVID-19 auf (RKI, 2021g). Es fehlt jedoch noch an wissenschaftlicher Evidenz um einzuschätzen, in welchem Maß Impfstoffe die Übertragbarkeit des Virus reduzieren (RKI, 2021g). Aufgrund von neuen Virusmutationen könnte eine breitere Bevölkerungsimmunität als die im Frühjahr genannten 70 % notwendig sein (Moore et al., 2021). Auch die Frage, über welchen Zeitraum eine geimpfte Person vor einer COVID-19-Erkrankung geschützt ist, ist derzeit noch ungeklärt (RKI, 2021e). All diese Aspekte könnten es durchaus begründen, weiterhin ein möglichst diversifiziertes Portfolio an Impfstoffen zu beschaffen und entsprechend höhere Liefermengen vertraglich abzusichern. Dies betrifft insbesondere auch Impfstoffvarianten, die mit Blick auf eine gute Wirksamkeit zum Schutz vor neuen Virusvarianten entwickelt werden.

Abbildung 4

**Zahl der durchgeführten COVID-19-Impfungen je 100 Einwohner:innen, weltweit[1]**

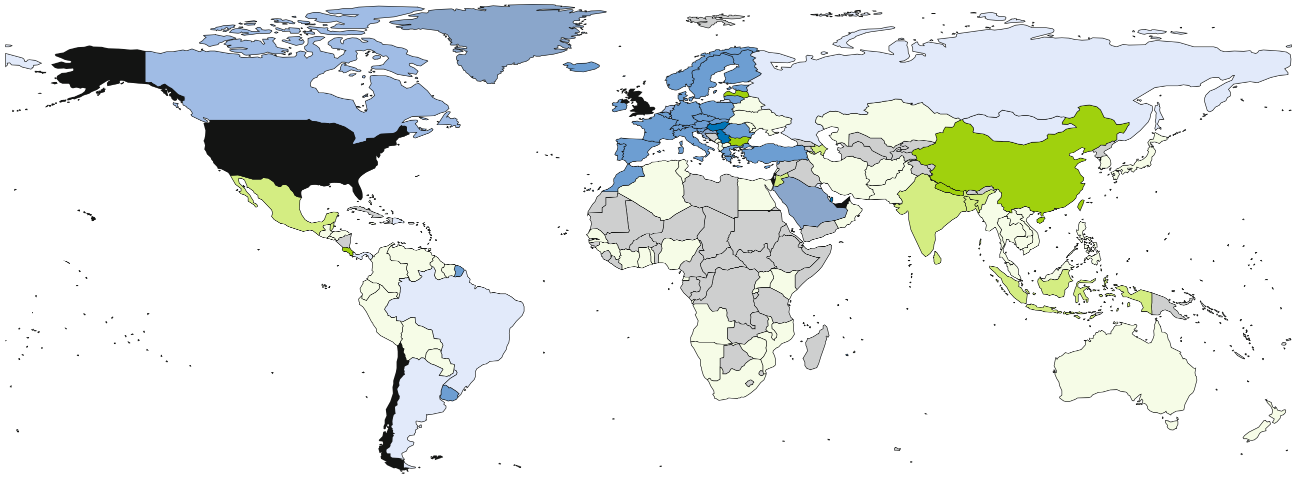

Zahl der durchgeführten COVID-19-Impfungen je 100 Einwohner:innen:

0 bis 3 | > 3 bis 5 | > 5 bis 7 | > 7 bis 9 | > 9 bis 11 | > 11 bis 13 | > 13 bis 20 | > 20 bis 35 | > 35

Keine Werte verfügbar

[1] Jede Impfung wird als Einzeldosis gezählt und entspricht möglicherweise nicht der Gesamtzahl der geimpften Personen, wenn diese z. B. bereits mehrere Dosen erhalten haben (Stand 26. März 2021).

Quellen: EuroGeographics bezüglich der Verwaltungsgrenzen; Our World in Data.

Um eine hohe Bevölkerungsimmunität zu erreichen und da aktuell die Fallzahlen speziell bei Kindern und Jugendlichen stark ansteigen (RKI, 2021f), ist es wichtig, auch diese Altersgruppe gegen COVID-19 zu impfen. Für Kinder und Jugendliche gibt es aufgrund eines fehlenden Impfstoffs zudem derzeit noch Unklarheit bezüglich der Perspektiven auf ein Ende der gesundheitspolitischen Einschränkungen. Deshalb sollte in die Entwicklung von neuen Impfstoffen gegen COVID-19 für diese Altersgruppe sowie in Impfstudien zur Erprobung der existierenden Vakzine bei Kindern und Jugendlichen umfangreich investiert werden, damit eine Zulassung zeitnah erfolgen kann. Bisher ist nur das Vakzin von BioNTech/Pfizer in der EU für Jugendliche ab 16 Jahren zugelassen. Zudem hat der Pharmakonzern bereits Impfstofftests bei Kindern unter 12 Jahren begonnen, mit der Aussicht auf eine Ausweitung der Impfung auf diese Altersgruppe bis Anfang 2022 (Pfizer, 2021; Reuters, 2021). Auch Moderna erprobt aktuell im Rahmen der KidCove-Studie die Wirksamkeit seines Vakzins auch an jüngeren Kindern (Moderna, 2021).

Um sich gegen das Risiko zukünftiger Pandemien in Europa abzusichern, ist eine gemeinsame europäische Strategie zum Schutz vor Unterbrechungen der Lieferketten – etwa von medizinischen Gütern wie Arzneimitteln, Impfstoffen oder medizinischen Geräten – sowie eine Stärkung der EU im Hinblick auf grenzüberschreitende Gesundheitsbedrohungen angezeigt. Mit der Planung einer EU-Behörde für die Krisenvorsorge und -reaktion bei gesundheitlichen Notlagen (HERA) von der EU-Kommission wurde hierfür ein wichtiger Grundstein gelegt (EU-Kommission, 2021k). Bei der Umsetzung sollte darauf geachtet werden, dass der Koordinierungsbedarf die Reaktionsfähigkeit nicht behindert.

Als Grundlage für HERA hat die EU-Kommission bereits im Februar 2021 einen europäischen Bereitschaftsplan zur Vorsorge gegen biologische Gefahren im Zusammenhang mit COVID-19-Varianten (HERA-Inkubator) erstellt (EU-Kommission, 2021f, 2021g). Ziel ist es, dass Forschung, Biotechunternehmen, Pharmahersteller und Behörden aus der EU und der ganzen Welt zusammenarbeiten. Die darin enthaltenen Sofortmaßnahmen sollen Anreize zur Herstellung von neuen und angepassten Impfstoffen schaffen, die Entwicklung wirksamer Impfstoffe gegen Virusvarianten vorantreiben, Zulassungsverfahren für diese Impfstoffe beschleunigen – etwa eine schnellere Zulassung angepasster Impfstoffe sowie Unterstützung der Forschung bei der Einbeziehung von Kindern in klinische Prüfungen – und europäische industrielle Produktionskapazitäten steigern. Mit Blick auf den zukünftigen Bedarf an Impfstoffen ist ein Aufbau langfristiger Partnerschaften mit den Pharmaherstellern und die Mobilisierung von umfassenden Ressourcen zielführend. Aufgrund der hohen Unsicherheit, ob und in welchem Umfang Neu- oder Auffrischungsimpfungen gegen COVID-19 in Zukunft erforderlich sein werden, könnten private Investitionsanstrengungen der Unternehmen ineffizient niedrig bleiben.

Die Impfstoffverfügbarkeit ist derzeit weltweit extrem ungleich verteilt. Ende Januar 2021 wurden 75 % der bisher verbrauchten Dosen in nur zehn Ländern verimpft (WHO, 2021). Im weltweiten Vergleich zeigt sich vor allem, dass die Impfkampagnen in den fortgeschrittenen Volkswirtschaften und einigen Schwellenländern unterschiedlich schnell voranschreiten (vgl. Abbildung 4). Dabei ist die Impfstoffversorgung der ärmeren Länder im Interesse der Industriestaaten. Die Wohlfahrtsgewinne massiver Investitionen in den globalen Impffortschritt dürften die Kosten, die eine ungleiche Verteilung des Impfstoffs für die entwickelten Volkswirtschaften zur Folge hat, um ein Vielfaches übersteigen (Castillo et al., 2021; Çakmaklı et al., 2021). Zirkuliert zudem das Virus in einigen Teilen der Welt weiterhin unkontrolliert, steigt das Risiko, dass die Virusmutationen zukünftig weitere Varianten entwickeln und dass die verfügbaren bzw. teilweise bereits verabreichten Impfstoffe nicht mehr wirksam sind (Kupferschmidt, 2021). Vor diesem Hintergrund ist eine umfassende Unterstützung von Initiativen wie COVAX, die Ländern unabhängig von ihrer Kaufkraft zügigen Zugang zu Impfstoffen gegen COVID-19 verschaffen will, positiv zu bewerten und sollte von der EU und den Mitgliedstaaten konsequent verfolgt werden.

**Title:** ***Opportunities and Risks of Vaccination Progress***

**JEL Classification:** *H4, H12, H51, I18*
